# High-Volume Liposuction in Lipedema Patients: Effects on Serum Vitamin D

**DOI:** 10.3390/jcm13102846

**Published:** 2024-05-11

**Authors:** Tonatiuh Flores, Celina Kerschbaumer, Florian J. Jaklin, Christina Glisic, Hugo Sabitzer, Jakob Nedomansky, Peter Wolf, Michael Weber, Konstantin D. Bergmeister, Klaus F. Schrögendorfer

**Affiliations:** 1 Karl Landsteiner University of Health Sciences, Dr. Karl-Dorrek-Straße 30, 3500 Krems, Austria; 51807592@edu.kl.ac.at (C.K.); christina.glisisc@stpoelten.lknoe.at (C.G.); hugo.sabitzer@stpoelten.lknoe.at (H.S.); jakob.nedomansky@stpoelten.lknoe.at (J.N.); michael.weber@kl.ac.at (M.W.); konstantin.bergmeister@stpoelten.lknoe.at (K.D.B.); klaus.schroegendorfer@stpoelten.lknoe.at (K.F.S.); 2 Clinical Department of Plastic, Aesthetic and Reconstructive Surgery, University Clinic of St. Poelten, 3100 St. Poelten, Austria; 3 Clinical Laboratory for Bionic Extremity Reconstruction, University Clinic for Plastic, Reconstructive and Aesthetic Surgery, Medical University of Vienna, 1090 Vienna, Austria; florian.jaklin@meduniwien.ac.at; 4 Division of Endocrinology and Metabolism, Department of Internal Medicine III, Medical University of Vienna, 1090 Vienna, Austria; peter.wolf@meduniwien.ac.at

**Keywords:** liposuction, lipedema, vitamin D, BMI

## Abstract

**Background**: Lipedema is a subcutaneous adipose tissue disorder characterized by increased pathological adipocytes mainly in the extremities. Vitamin D is stored in adipocytes, and serum levels inversely correlate with BMI. As adipocytes are removed during liposuction, lipedema patients might be prone to further substantial vitamin D loss while their levels are already decreased. Therefore, we examined the effect of liposuction on perioperative serum 25-hydroxyvitamin D levels. **Methods**: In patients undergoing lipedema liposuction, blood samples were obtained pre- and postoperatively. Statistical analyses were performed to correlate the volume of lipoaspirate, patients’ BMI and number of sessions to vitamin D levels. **Results**: Overall, 213 patients were analyzed. Mean liposuction volume was 6615.33 ± 3884.25 mL, mean BMI was 32.18 ± 7.26 kg/m^2^. mean preoperative vitamin D levels were 30.1 ± 14.45 ng/mL (borderline deficient according to the endocrine society) and mean postoperative vitamin D levels were 21.91 ± 9.18 ng/mL (deficient). A significant decrease in serum vitamin D was seen in our patients (*p* < 0.001) of mean 7.83 ng/mL. The amount of vitamin D loss was not associated with BMI or aspiration volume in our patients (*p* > 0.05). Interestingly, vitamin D dynamics showed a steady drop regardless of volume aspirated or preoperative levels. **Conclusions**: Many lipedema patients have low vitamin D levels preoperatively. Liposuction significantly reduced these levels additionally, regardless of aspirated volume or BMI. However, vitamin D loss was constant and predictable; thus, patients at risk are easily identified. Overall, lipedema patients undergoing liposuction are prone to vitamin D deficiency, and the long-term effects in this population are currently unknown.

## 1. Introduction

Lipedema is a subcutaneous adipose tissue disorder almost solely affecting women [1,2]. Patients mainly suffer from painful localized fat deposition in the extremities with consecutive restrictions in daily life [2,3,4]. Due to its complex etiology and variable clinical manifestations, it presents as a multifaceted challenge for modern surgery. A vast number of comorbidities are associated with lipedema, such as hypertension, depression or increased BMI [5,6]. Vitamin D is stored in fat tissue, and due to its inverse correlation with BMI status and body fat, vitamin D serum deficiency can frequently be seen in lipedema patients [5,7,8,9,10,11,12,13,14,15,16]. It is the most common deficiency in obese patients worldwide with a prevalence of 80–90% [9,17,18,19,20,21,22,23,24,25].

Besides their painful nature, lipedematous adipocytes are resistant to diets and bariatric surgery [6,26,27]. Despite growing recognition, effective treatment modalities for lipedema remain limited. Therefore, liposuction has emerged as the only suitable procedure for managing lipedema-related symptoms, offering symptomatic relief to reduce patients’ burden and enhance their quality of life [25,26].

To our knowledge, this is the first study investigating the relationship of liposuction and vitamin D serum levels in lipedema patients. Hence, in this study we seek to critically examine the implications of vitamin D deficiency in lipedema patients after liposuction, elucidating the potential challenges and proposing strategies for mitigating adverse outcomes. We aimed to analyze perioperative vitamin D alternations in lipedema patients undergoing liposuction.

## 2. Materials and Methods

### 2.1. Study Design and Patient Analysis

In this study we analyzed pre- and postoperative vitamin D serum levels in lipedema patients undergoing liposuction at the Clinical Department for Plastic, Aesthetic and Reconstructive Surgery at the University Hospital St. Poelten, between 1 January 2018 and 31 December 2022. The study was conducted as a retrospective single center study. Ethical approval was obtained from the local institutional review board at the Karl Landsteiner University of Health Sciences Krems (reference number: ECS 1041/2021). Analyzed factors included the patients’ age at surgery, BMI, volume of lipoaspiration, pre- and postoperative serum vitamin D levels, localization of treated area (upper and lower extremities) and liposuction sessions (one, two or three sessions). 

### 2.2. Operative Procedure

**Figure 1:**
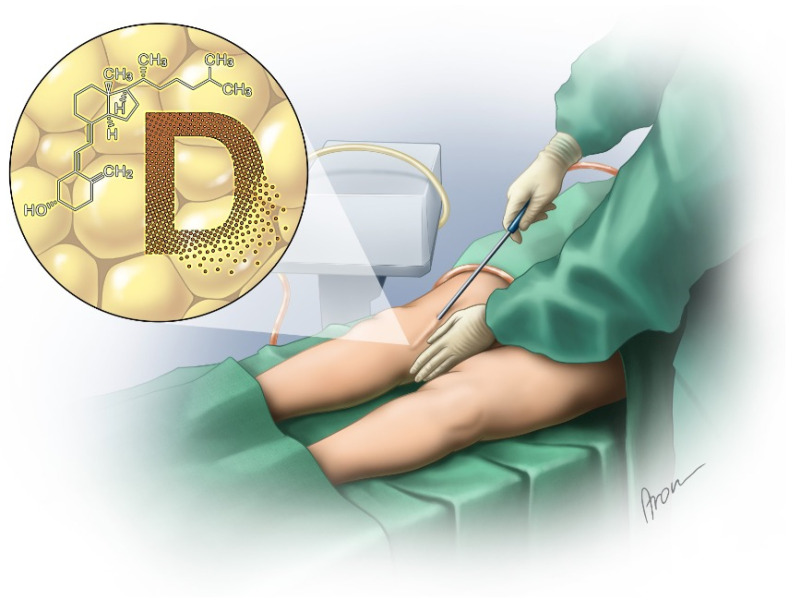
Illustration demonstrating the depletion of vitamin D during liposuction.

At our department, liposuction is performed under general anesthesia using tumescent technique. Patients are examined and marked preoperatively while standing to assess areas to be treated. All patients receive intravenous antibiotic shielding with either 2.2 g of amoxicillin/clavulanic acid combination (Curam^®^, Sandoz GmbH, 6250 Kundl, Austria) or 600 mg of clindamycin (Dalacin^®^, Fareva Amboise Zone Industrielle, Routes des Industries 29, 37530 Pocé-sur-Cisse, France) in case of penicillin allergy. Antibiotic administration is given 30 min before surgical incision and is continued for one week postoperatively. Patients receive a modified Klein’s solution with 1.000 mL Ringer’s lactate (Ringer lactate^®^, Fresenius Kabi, Rue du Rempart 6, 27400 Louviers, France) containing 1 mL of 1:1.000 epinephrin (Suprarenin^®^ Sanofi-Aventis GmbH, 65926 Frankfurt am Main, Germany). The solution is infiltrated with specialized infiltration cannulas through small stab incisions at strategically placed locations using a number 11 blade, which can easily be camouflaged postoperatively by the patient’s clothing (e.g., the groin). After an indwelling time of approximately 15 min for the tumescent solution to set, vibration-assisted liposuction (VAL) is performed using Moeller’s liposuction device (Moeller Vibrasat Pro, Moeller medical^®^ GmbH, Wasserkuppenstraße 29-31, 36043 Fulda, Germany) with 3 and/or 4 mm multiport cannulas (multiport rapid extraction cannula, Moeller Medical^®^ GmbH, Wasserkuppenstraße 29-31, 36043 Fulda, Germany) (Figure 1). Incisions are not sutured and are solely covered with plasters after antiseptic irrigation with Octenisept^®^ (Schülke & Mayr GmbH, Robert-Koch-Straße 2, 22851, Norderstedt, Germany) and Skinsept^®^ (Ecolab Germany GmbH, Ecolab-Allee 1, 40789 Monheim am Rhein, Germany). Compression garments are installed immediately postoperatively in the operating room. Compression garments are worn day and night for three months postoperatively. Patients receive antithrombotic shielding using low molecular heparin for 10 to 30 days postoperatively. 

### 2.3. Blood Sampling

Blood samples were collected preoperatively at a maximum of one week prior to surgery and analyzed for serum 25-hydroxyvitamin D levels at our clinical institute of laboratory medicine. The vacutainers used for vitamin D sampling were BD Vacutainer^®^ with stabilizing gel (Fischer Scientific GmbH, Im Heiligen Feld 17, 58239 Schwerte, Germany). Vitamin D components were separated using Elecsys Vitamin D total III Cobas^®^ (Roche Diagnostics GmbH, Sandhofer Straße 116, 68305 Mannheim, Germany). All samples were retrieved and processed using the same instruments. Sample results were digitally stored at the hospital’s data working space adhering to Austrian regulations for data protection. Postoperative sample collection was performed on the first postoperative day. Serum vitamin D levels below 30 ng/mL, according to the Endocrine Society were indicated as deficiency [28]. 

### 2.4. Statistics and Data Management

The endpoint of our analyses was to assess the alteration of vitamin D serum levels after liposuction and high-volume liposuction in lipedema patients. All data were reported anonymously. Data protection management complied with Austrian legislation. Data collection and processing were performed with Microsoft Excel (Software Version 2021, Microsoft Corp., One Microsoft Way, Redmond, 98052 Washington, DC, USA), and statistical analyses were performed using IBM SPSS Statistics version 26 (©IBM, Armonk, NY, USA). Nominal data were described using absolute frequencies and percentages. For metric data, mean and standard deviation were indicated. To correlate the volume of lipoaspirate and patients’ BMI to vitamin D alterations, correlation analyses using Spearman’s rho test were performed. Further, paired *t*-test analyses were conducted. Two-sided *p* ≤ 0.05 was regarded as statistically significant. To analyze the decrease in vitamin D levels regarding liposuction sessions, analysis of variance (ANOVA) was used.

## 3. Results

### 3.1. Demographics

**Figure 2:**
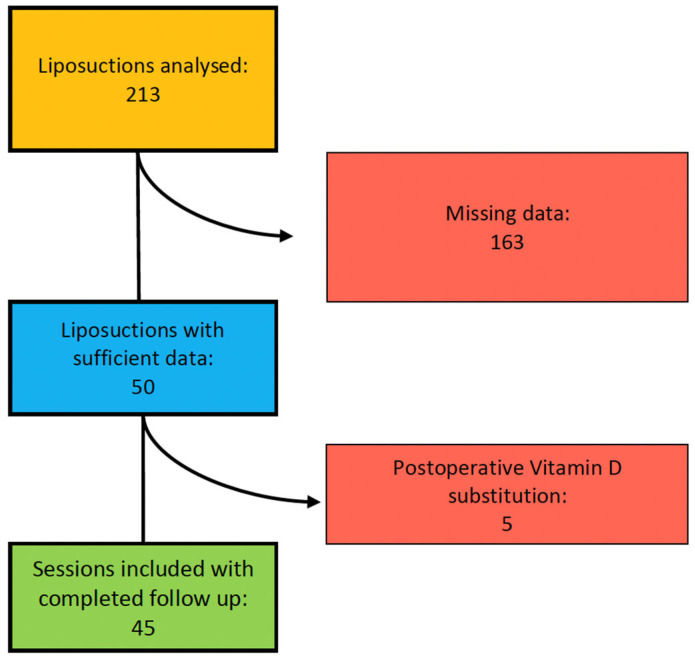
Organigram of patient selection for study inclusion.

In total, 213 liposuctions in 100 patients suffering from lipedema were identified during the study period. Thereof, 163 liposuctions in 61 patients were excluded due to missing data (Figure 2). 

**Figure 3:**
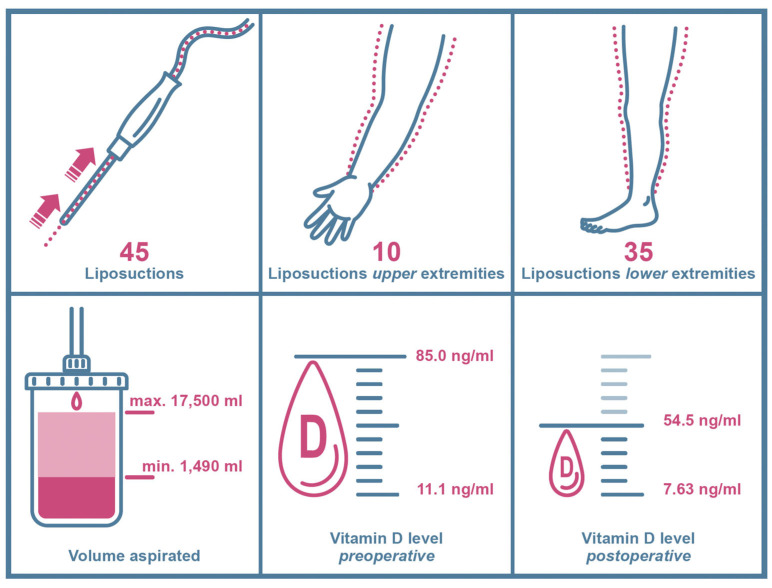
Key data chart of included study patients and clinical findings. Arrows and dotted lines in the upper left window show the suction path (arrows) of lipoaspirate (dotted lines) during liposuction.

Additionally, five liposuctions in three patients were excluded because of self-supplied postoperative vitamin D substitution. Consequently, 45 liposuctions in 36 patients met our criteria and were included in our study. We analyzed 35 liposuctions on lower extremities and 10 liposuctions on upper extremities (Figure 3). All patients included were Caucasian women and did not expose themselves excessively to the sun or substitute vitamin D independently according to the anamnesis. 

Mean patient age was 38.11 ± 13.74 years overall, ranging from 19 years as the youngest to 71 years as the oldest at time of surgery (Table 1). Mean BMI was 32.18 ± 7.26 kg/m^2^, varying from 21.7 kg/m^2^ to 53.1 kg/m^2^ (Table 1). Mean volume aspirated was 6615.33 ± 3884.253 mL, with a minimum of 1490 mL and maximum of 17,500 mL in one session (Table 1). Patients were further divided into two groups as higher volumes of liposuction mainly occur in the lower extremities: patients undergoing liposuction on upper extremities and patients undergoing liposuction on lower extremities. 

### 3.2. Liposuction of Upper Extremities

Patients’ mean age in this group was 39.0 ± 15.74 years, ranging from 20 years to 57 years. Mean BMI in the upper extremity group was 29.21 ± 3.96 kg/m^2^, ranging from 24.2 kg/m^2^ to 36.4 kg/m^2^. Mean volume aspirated was 3845 mL ± 3884.25 mL with a minimum of 1800 mL and a maximum of 7600 mL in one session (Table 1).

### 3.3. Liposuction of Lower Extremities

In the lower extremity group, patients’ mean age was 37.86 ± 13.36, ranging from 19 to 71 years. Mean BMI was 33.03 ± 7.79 kg/m^2^, ranging from 21.7 kg/m^2^ to 53.1 kg/m^2^. Mean volume aspirated was 7406.86 ± 3997.92 mL with a minimum of 1490 mL and a maximum of 17,500 mL in one session (Table 1). 

### 3.4. Vitamin D Serum Levels

**Figure 4:**
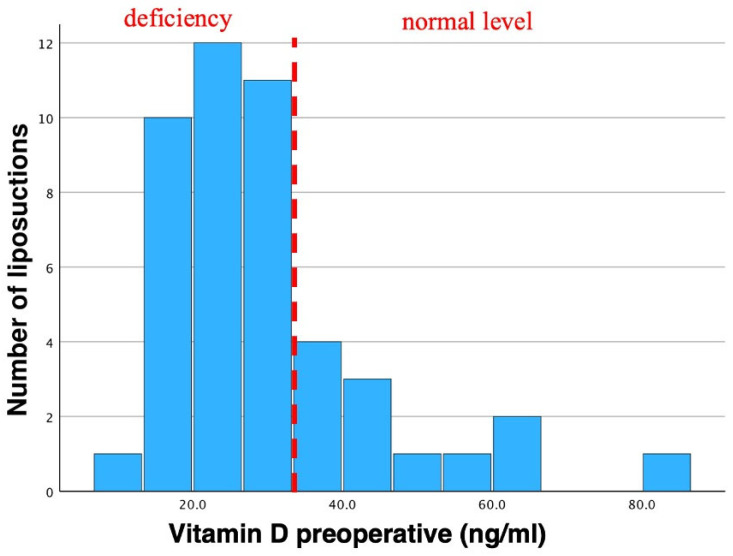
Histogram of preoperative vitamin D. Mean vitamin D levels were 30.1 ng/mL in total. Std. was 14.45 ng/mL (N = 46). Preoperative vitamin D insufficiency (according to the endocrine society) can be observed as most bars are shifted to the left. This vitamin D insufficiency is often seen in lipedema patients. Dotted line indicates the threshold of vitamin D deficiency to non-deficiency in preoperative patients.

In total, mean preoperative vitamin D levels were 30.1 ± 14.45 ng/mL, ranging from 11.1 ng/mL to 85.0 ng/mL (Table 1, Figure 4). 

**Figure 5:**
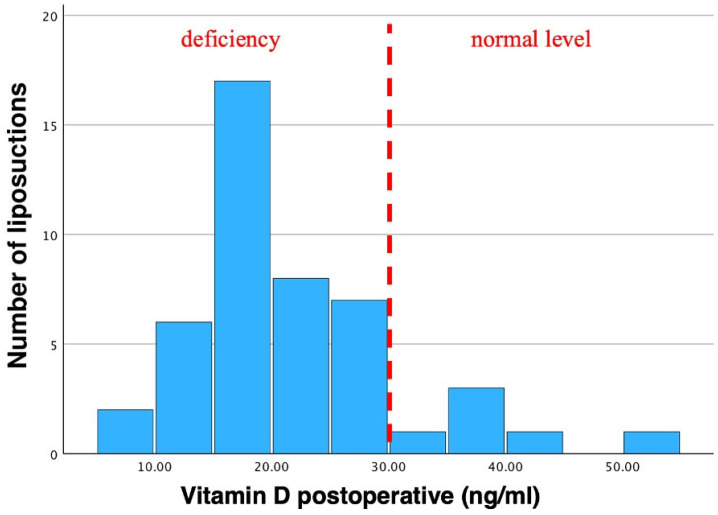
Histogram of postoperative vitamin D levels. Mean vitamin D was 21.91 ng/mL. Std. was 9.18 ng/mL (N = 46). A vitamin D insufficiency can clearly be seen in postoperative values as the bars are shifted to the left. Pre-existing vitamin D insufficiencies are further aggravated through liposuction. Dotted line indicates the threshold of vitamin D deficiency to non-deficiency in postoperative patients.

Mean postoperative vitamin D levels were 21.91 ± 9.18 ng/mL, ranging from 7.63 ng/mL to 54.5 ng/mL (Table 1, Figure 5).

In total, 29 patients showed preoperative vitamin D levels below 30 ng/mL. Postoperatively, 40 patients showed vitamin D levels below 30 ng/mL. None of our patients with preoperative vitamin D levels below 30 ng/mL showed any clinical sign of deficiencies. Vitamin D was not substituted in our cohort, either pre- or postoperatively.

### 3.5. Vitamin D Serum Levels of Liposuction of Upper Extremities

In this group, mean preoperative vitamin D levels were 33.24 ± 14.66 ng/mL, displaying a range from 15.8 ng/mL to 64.3 ng/mL. Mean postoperative vitamin D levels were 25.85 ± 12.44 ng/mL, measuring from 13.7 ng/mL to 54.5 ng/mL (Table 1, Figure 3).

### 3.6. Vitamin D Serum Levels of Liposuction of Lower Extremities

Mean preoperative vitamin D levels were 29.20 ± 14.47 ng/mL, ranging from 11.1 ng/mL to 85.0 ng/mL in patients treated at lower extremities. Mean postoperative vitamin D levels were 20.79 ± 7.89 ng/mL, ranging from 7.63 ng/mL to 42.5 ng/mL (Table 1, Figure 3).

### 3.7. Correlation Analysis

We correlated patients’ BMI with the amount of ml aspirated during liposuction, expecting a higher BMI drop at higher liposuction volumes. Using Spearman’s rho test for rank correlation, our analyses showed no significant correlation, either in absolute numbers (*p* = 0.006) or in relative numbers (*p* = 1.97) (Table 2), demonstrating that the absolute amount of volume reduced does not interfere with the patients’ BMI. 

Investigating the patients’ vitamin D serum levels pre- and postoperatively, we found a statistically significant decrease regarding vitamin D levels using a paired *t*-test (*p* < 0.001, Table 3). These findings were both significant overall and between the different groups (*p* < 0.001, Table 3). Since our data set turned out not to be normally distributed (outliers included in our data set), we additionally conducted the according non-parametric tests. Nonetheless, our data were still significant, additionally supporting our statistical findings (*p* < 0.001; labeled in red, Table 3).

This demonstrates that high-volume liposuction has a significant impact on postoperative vitamin D level changes. The abovementioned findings were also significant when analyzing areas treated separately (upper extremities and lower extremities). Hence, the decrease in vitamin D after liposuction was significant, no matter of liposuction location (*p* < 0.001). 

The abovementioned findings were also significant when analyzing areas treated separately (upper extremities and lower extremities, Table 4). Hence, the decrease in vitamin D after liposuction was significant, no matter the area treated (*p* < 0.001, Table 4). Again, *p*-values of non-perimetric tests for non-normal distribution were also significant (*p* = 0.005 in upper extremities and *p* < 0.001 in lower extremities; labeled in red, Table 4).

Interestingly, after performing ANOVA (analysis of variance) for correlation of vitamin D level changes and the volume aspirated, we did not find any significant correlation (*p* = 0.906 in absolute numbers, and *p* = 0.451 in relative numbers, Table 5). This finding was also seen in non-perimetric testing (*p* = 0.481 in absolute numbers, and *p* = 0.128 in relative numbers; labeled in red, Table 5). These findings were consistent throughout the session of liposuction.

The findings demonstrate that no matter the volume removed during liposuction, vitamin D levels did not drop concordantly. Our analyses rather showed a non-correlation between the decrease in vitamin D after liposuction and the volume aspirated, hence demonstrating a stable drop in vitamin D between a mean of 6.86 ng/mL and 8.81 ng/mL (mean 7.83 ng/mL) no matter the volume of lipoaspirate.

## 4. Discussion

Many studies have been conducted to analyze serum levels of vitamin D after diets or bariatric surgery [10,11,12,29,30,31,32,33], yet none have investigated the alteration in vitamin D levels after liposuction in lipedema patients. To our knowledge, this is the first study investigating the correlation of vitamin D serum levels after liposuction.

Adipose tissue plays a significant role in energy supply and distribution and is essential in storing fat-soluble vitamins, such as vitamin D. Its bioactivity includes the reduction in inflammatory processes, neuromuscular regulation as well as the absorption of calcium, an essential mineral in osteosynthesis [29,34]. Vitamin D deficiency can lead to diminished immune responses, muscle weakness, osteoporosis and increased fracture rates [19,22,23,34,35,36,37]. Approximately 1 billion people (developing and developed countries) suffer from vitamin D deficiency, consequently making it a global public health issue [20,21,37,38]. It is the most common deficiency in obese patients worldwide [9,17,18,19,20,21,22,23,24,25]. Vitamin D is normally synthesized through the skin, yet obese patients have significantly lower levels in their blood stream compared to non-obese patients, despite having increased body surfaces [8,19,39]. This results from the inverse correlation of vitamin D and the patients’ BMI, as more adipocytes store more vitamin D [5,7,8,9,10,11,12,13,14,15], thus, leading to serum deficiency. Since most lipedema patients have elevated BMI due to pathologically engorged adipocyte, this cohort often shows vitamin D deficiency as well. These patients not only display low vitamin D serum levels due to its inverse correlation to BMI but also because engorged and inflamed adipocytes in lipedema traps vitamin D [14,40,41,42]. This bidirectional relationship between vitamin D deficiency and elevated BMI with low serum vitamin D levels is also evident in our study.

Several studies have demonstrated that weight loss by lifestyle changes or bariatric surgeries increases vitamin D levels [8,10,11,29,43,44]. Nevertheless, this effect was not detected in our study after liposuction so far. Contrarily, our results showed a significant decrease in postoperative vitamin D levels after treatment (Figure 5). 

Since liposuction is the gold standard in treating lipedema, patients already suffering from vitamin D deficiency are at risk of further vitamin D loss. The lack of vitamin D has already been linked to entailing chronic cellular stress, which can be seen in lipedema patients [40,42,45]. By further diminishing vitamin D levels after liposuction, patients experiencing lipedema further maintain oxidative stress, resulting in sustained lipedema symptoms [14,42,45]. Thus, lipedema symptoms might not ameliorate after treatment, potentially aggravating their symptoms. Although the postoperative 25-hydroxyvitamin D decrease did not drop concordantly with the volume aspirated, its decrease was significant in our findings. Therefore, lipedema patients ought to be screened for vitamin D deficiency and, if needed, it should be substituted to prevent further sequelae, such as osteopenia or osteoporosis.

To personalize liposuction for lipedema patients, preoperative assessment of vitamin D levels in concordance with the estimated amount of lipoaspirate is needed. Thereby, vitamin D levels can be improved before surgery and ensure patients face the operation in an optimized manner. Parenthetically, high-volume liposuction needs to be separated into several sessions to obviate excessive vitamin D loss postoperatively. Vitamin D levels in addition are to be monitored precisely between sessions to prevent patients from experiencing vitamin D deficiency in between treatment sessions. Interdisciplinary collaboration involving endocrinologists and nutritionists is essential for implementing patient-tailored strategies. Perioperative optimization of nutrition management or the administration of supplements to individually address the needs of lipedema patients is therefore favored to guarantee long-term patient safety.

## 5. Conclusions

Although liposuction is a relatively safe procedure, its aftereffects are not to be neglected. To protect this patient cohort from further long-term sequelae and to sufficiently relieve patients’ burden, perioperative improvement of treatment modalities is necessary. By addressing vitamin D deficiency comprehensively, healthcare providers can enhance the efficacy of liposuction as a therapeutic tool for managing lipedema. To reduce postoperative vitamin D loss in lipedema patients, high-volume liposuction ought to be stratified and personalized to each patient individually for optimized vitamin D preservation. Therefore, lipedema patients might not suffer further comorbidities related to their underlying disease after treatment. Despite the significant findings in our research, our study faces a few limitations. Postoperative vitamin D levels should be monitored over a longer period. Also, the storage behavior and characteristics of lipedema adipocytes ought to be investigated through controlled histological analyses. Additional cross-section studies are needed for further detection of this underacknowledged threat.

## Figures and Tables

**Table 1:** Baseline characteristics of patients. Significantly lower vitamin D serum levels can be observed postoperatively.

Patient Characteristics	Total	Upper Extremities	Lower Extremities
Number	**45**	**10**	**35**
Age—years mean (standard deviation)	38.11 (std.: 13.74)	39.0 (std.: 15.74)	37.86 (std.: 13.36)
min.–max.	19–71	20–57	19–71
BMI—kg/m^2^ mean (standard deviation)	32.18 (std.: 7.26)	29.210 (std.: 3.96)	33.029 (std.: 7.79)
min.–max.	21.7–53.1	24.2–36.4	21.7–53.1
Vit D pre—ng/mL mean (standard deviation)	30.1 (std.: 14.45)	33.240 (std.: 14.66)	29.203 (std.: 14.47)
min.–max.	11.1–85.0	15.8–64.3	11.1–85.0
Vit D post—ng/mL mean (standard deviation)	21.914 (std.: 9.18)	25.85 (std.: 12.44)	20.7903 (std.: 7.89)
min.–max.	7.63–54.5	13.7–54.5	7.63–42.5
Volume—mL mean (standard deviation)	6615.33 (std.: 3884.25)	3.845 (std.: 3884.25)	7406.86 (std.: 3997.91)
min.–max.	1490–17,500	1800–7600	1490–17,500

**Table 2:** Spearman’s rho test for rank correlation demonstrating no significant correlation between the volume of fat removed and the decrease in patients’ BMI (*p*-values > 0.05). Regardless of the volume aspirated, the difference in pre- and postoperative BMI did not show significant changes. This finding is displayed in absolute and relative numbers within this table.

Spearman’s Rho Correlation of Volume Aspirated and BMI				
			**Volume Aspirated**	**BMI**
**Spearman’s Rho**	BMI	Correlation coefficient	0.632	
		Sig. (2-sided)	<0.001	
		N	45	
	Absolute decline	Correlation coefficient	0.006	0.052
		Sig. (2-sided)	0.969	0.732
		N	45	45
	Relative decline	Correlation coefficient	0.197	0.059
		Sig. (2-sided)	0.195	0.698
		N	45	45

**Table 3:** *t*-Test analysis showing the significant correlation between the measured vitamin D serum levels pre- and postoperatively (*p* < 0.001).

*t*-Test					
		**Mean**	**N**	**Std.-Deviation**	***p*-Value**
**Pairing**	**Vit. D pre**	30.10	45	14.45	<0.001/<0.001
	**Vit. D post**	21.91	45	9.18	

**Table 4:** *t*-Test analysis on account of vitamin D decrease after liposuction in upper and lower extremities, demonstrating the statistical significance *p* < 0.001 in both groups (arms and legs). *p*-Values were still significant after non-perimetric testing (*p* < 0.001 and *p* = 0.005; labeled in red).

*t*-Test						
**Localization**			**Mean**	**N**	**Std. Deviation**	***p*-Value**
Legs		Vit. D pre	29.203	35	14.4755	<0.001/<0.001
		Vit. D post	20.7903	35	7.89464	
Arms		Vit. D pre	33.240	10	14.6677	<0.001/0.005
		Vit. D post	25.8500	10	12.44412	

**Table 5:** Analysis of variance (ANOVA) of volume aspirated during liposuction and decrease in serum vitamin D levels. Here, no significant correlation can be observed (*p* = 0.906 in absolute numbers, and *p* = 0.451 in relative numbers), More likely, our ANOVA analysis shows a non-correlation, concluding that no matter the amount of volume aspirated, vitamin D levels do not drop concordantly. Rather, a stable decrease in vitamin D can be seen regardless of volume of lipoaspirate.

Analysis of Variance (ANOVA)							
		N	Mean	Std. Deviation	*p*-Value	95% Mean Confidence Interval	
						Lower Limit	Upper Limit
Absolute decrease	1	22	8.81	13.16	0.906/0.481	2.98	14.65
	2	20	7.68	3.58		6.00	9.36
	3	3	6.86	8.01		−13.04	26.77
	total	45	8.18	9.57		5.30	11.06
Relative decrease	1	22	23.03	17.35	0.451/0.128	15.34	30.73
	2	20	27.19	10.53		22.26	32.12
	3	3	17.57	11.76		−11.64	46.80
	total	45	24.52	14.33		20.21	28.83

## Data Availability

All the data analyzed during the current study are available from the corresponding author on reasonable request.
